# Prevalence of hepatitis E virus in thalassemia patients with hepatitis C in Tehran, Iran

**Published:** 2019-12

**Authors:** Najmeh Dalvand, Azadeh Dalvand, Zohreh Sharifi, Seyed Masoud Hosseini

**Affiliations:** 1 Blood Transfusion Research Center, High Institute for Research and Education in Transfusion Medicine, Tehran, Iran; 2 Department of Microbiology & Microbial Biotechnology, Faculty of Life Sciences & Biotechnology, Shahid Beheshti University, Tehran, Iran

**Keywords:** Hepatitis E virus, Thalassemia, Hepatitis C virus, Genotype

## Abstract

**Background and Objectives:**

HEV infection is predominantly spread via the fecal-oral route; however, due to the presence of HEV RNA in the serum of healthy blood donors, there is a possibility of the transmissibility of HEV infection through blood. Multi-transfused thalassemia patients are one of the high risk groups for blood borne viruses. In this study, we evaluated the prevalence of HEV antibodies and HEV-RNA in thalassemia patients with HCV infection.

**Materials and Methods:**

120 anti-HCV positive thalassemia patient serum samples from Tehran province during April–June 2019 were assessed for the presence of total anti-HEV antibodies using of HEV Ab ELISA kit. All serum samples were assayed by Nested RT-PCR to detect HEV-RNA.

**Results:**

The results of ELISA test showed that 2 out of 120 (1.67%) samples were positive for anti-HEV Ab. There was no statistically significant difference between anti-HEV antibody prevalence rate and sex, age and other risk factors. None of 120 (0.00%) samples were positive for HEV-RNA by Nested RT-PCR.

**Conclusion:**

Seroprevalence of HEV in our study group was 1.67% which is less than HEV seroprevalence rate in Iranian general population. Therefore, it can be conclude that transmission of HEV infection via blood transfusion seems to be uncommon in Iran and the fecal-oral route can be the predominant mode of transmission in Iran; however, more studies are required to confirm this issue.

## INTRODUCTION

Hepatitis E virus (HEV) is an established zoonotic agent with global distribution that is known as the fifth form of viral hepatitis causing infection in many developing countries in Asia and Africa and also, sporadic cases of acute hepatitis and jaundice in many industrialized countries (1, 2). HEV infection is predominantly spread via the fecal-oral route especially contaminated water with animal or human feces (3–5). The other modes of HEV transmission remain controversial (6). Overall, during the acute phase of the viremia period, IgM rise significantly for about 3–8 months. If HEV- RNA be detectable during this period, it can refer to current infection. Afterwards, IgM becomes undetectable and IgG level has an upward trend and remains unchanged for years. In this case, in the absence of IgM and HEV- RNA, the presence of IgG can be as indicative of past infection (7). Transmission of HEV via blood transfusion has been suggested in several studies (8). Performed studies in Japan, UK, France and Saudi Arabia have confirmed the possibility of the transfusion transmissibility of HEV infection in multi-transfused patients (7, 9). It has been reported that HEV is transmissible via transfusion of positive anti-HEV IgM and negative anti-HEV IgG blood donors plasma to rhesus monkey (10). Transmission of HEV infection among family members has been seen in 1–2% of cases (4). HEV infection mostly affects children, young to middle aged adults (15–40 years old) and pregnant women (4, 7). Clinical symptoms of HEV infection may vary from severe hepatitis to asymptomatic anicteric and self-limiting illness (2, 11). The prevalence of HEV in general population is different in Iran from the highest rate in Mashhad city (14.2%) till the lowest rate in Sari city (2.3%) (12, 13). In a study reported that the prevalence of HEV in combination with other viral infections such as HIV patients was high (16.4%) in Shiraz city compared to the general population (14). Also, High prevalence of anti-HEV antibody in HBV (11.3%) and Hepatitis C virus (HCV) (7%)-infected patients compared to healthy blood donors (4.5%) in Tehran was reported (15). The seroprevalence of HEV among thalassemia patients with chronic hepatitis C was reported to be 1.6% (16).

One of the most important causes of chronic hepatitis, cirrhosis, and liver cancer is HCV infection. HCV is a blood borne infection and the most common route of HCV transmission is parenteral and contaminated blood through clinical intervention (17, 18). Approximately 170–200 million people were infected with HCV infection worldwide and each year there is 3 to 4 million of new case infection with this virus (18, 19). It has been reported that HCV infection prevalence is less than 1% in Iranian general population and between 0.12%–0.89% of the aforementioned population has anti-hepatitis C virus antibodies (7, 18, 20, 21). As previously mentioned by Fazel et al. co-infection of HEV in patients with chronic HCV leads to deterioration of liver disease and hepatic complications. Also, revealed that HCV viral load and progression of chronic hepatitis to fulminant hepatitis in co-infection of HCV and HEV was substantially higher compared to HCV- infected patients (11).

One of the high risk groups for HCV infection is multi-transfused individuals especially thalassemia patients due to the blood transfusion from asymptomatic HCV infected donors before HCV screening (21–23). Iran is located on the thalassemia belt with high thalassemia carrier rate and more than 25,000 registered transfusion dependent thalassemia (TDT) (21, 25, 26). Reports from Iran have showed that 18% of thalassemia patients have HCV infection and overall HCV prevalence is around 20% to 40% among Iranian TDT patients (5, 7, 23, 25).

Regular blood transfusion increase the possible transmission of blood-borne viruses such as HCV and also, accumulation of iron in different parts of the body can be the threat for TDT patient. In other words, iron overload and HCV infection increase the possibility of infection with other viral hepatitis such as HEV (24, 25). Therefore, in the present study, prevalence of HEV antibodies and HEV- RNA in thalassemia patients with HCV infection was evaluated; also, other risk factors of HEV transmission via blood transfusion in thalassemia patients with HCV infection were studied.

## MATERIALS AND METHODS

### Patients.

The cross-sectional study was carried out from April to June 2019. Participants were comprised of thalassemic patients who were anti-HCV positive. About 120 serum samples from thalassemic patients in Tehran province that were referred to the clinical laboratory of IBTO in 2019 were collected. All samples were stored in 1.5 mL Eppendorf tubes at −80°C until they were tested. Demographic features of participants were recorded by a questionnaire contained detailed questions regarding baseline data (sex, age, and marital status), and risk factors (a history of blood transfusion, hospital admission, and surgery). This study was approved by the committee of ethics and all participants were given informed consent. Sera of thalassemic patients that were reactive for anti-HCV by ELISA methods (Biomerieux, France) were included in this study. All serum samples were assessed for the presence of total anti- HEV antibodies (IgG and IgM) using of HEV Ab ELISA kit (Dia.pro, Diagnostic Bioprobes, Italy) according to the method described in manufacturers instructions. A total of 10 μl of each serum sample was used. At the end of the assay, optical density (OD value) of samples was read at 450 nm using an ELISA plate microtiter reader (Dynex Technologies). Cut- off was defined by use of the mean OD 450 nm value of the Negative Controls (NC) that were included in each assay. The numeric value of each sample calculated and results pinpointed as described in the manufacturers instructions. To confirm the initial results, all positive samples were retested with the same EIA kit.

### Molecular assays.

All serum samples were assayed by Nested RT- PCR to detect HEV RNA. In order to perform the test, total RNAs were extracted from the serum samples with the High Pure Viral Nucleic Acid kit (Roche) and the extracted RNAs were stored at −80°C. Afterwards, cDNA was synthesized in RNase- free tubes by means of Easy cDNA Reverse Transcription kit (Arya Tous, Mashhad, Iran) that contains all necessary components for conversion of total RNA to the cDNA. Consequently, to detect viral RNA, the Nested RT- PCR amplification was carried out with Emerald Max PCR master mix (Takara biokit, USA) containing 10 μl of 2× master mix, 1 μl of 10 μm forward primer and 1 μl of 10 μm reverse primer, 6 μl DDW and 2 μl of cDNA by thermocycler instruments (Palm Cycler, Corbett, Sydney, Australia) in two rounds with two sets of synthetic primer pairs (Bioneer, Daejeon, South Korea) for detection of the HEV genome. All of these primer pairs target the ORF2 region of the HEV. A part of the ORF2 sequence was amplified using the outer primer pairs (sense: 5′ GAG GCA GGC ACA ACT AAA GC 3′) and (antisense: 5′ AAG AAG GGG GGC ACA AG 3′) in the first round, and inner primer pairs (sense: 5′ GCA CCG GGT CGC TAT TTC 3′) and (antisense: 5′ TGA AGC TCA GCG ACA GTA GA 3′) in the second round (ORF2 PCR) (27).

Both PCR rounds were performed under the same condition according to the following cycling program; initial denaturation for 5 min at 95°C, 35 cycles of denaturation for 30s at 94°C, annealing for 30s at 51°C, and extension for 1min at 68°C with an additional 5 min at 72°C, and a final incubation for 3 min at 4°C. The size of the amplification products of the first- round PCR was 364 bp, and that of the second-round was 219 bp. The amplification products of second- round were electrophoresed on a 1.5% agarose gel containing 0.5× TBE buffer, stained with DNA Green Viewer, and photographed under UV light.

In each run, a negative serum sample and positive control were used during testing. Set-up PCR was performed using positive control, for this purpose, ORF2 complete sequence of the HEV genome was cloned in PGEM- B1 vector and ran as a positive control (Bioneer, Daejeon, South Korea). The Nested RT- PCR assay was carried out in duplicate, and reproducibility was confirmed.

### Statistical analysis.

All data were analyzed with SPSS (version 21) software. To evaluate the association between different factors, Chi- square analysis was performed. The level of significance was set at a P value of < 0.05.

## RESULTS

Total of 120 serum samples was assayed for HEV total antibodies (IgG and IgM). There were 42 (35.0%) males, 78 (65.0%) females; the mean age was 32. 26 ± 6.36 years range from 17 to 45 years old. On analyzing the samples with regards to the risk factors, 60 (50.00%) patients had a history of surgery, 55 (45.83%) patients had a history of hospital admission and 79 (65.83%) patients had a history of blood trans-fusion. Some patients had more than one the risk factors (Table 1).

**Table 1.** Prevalence of anti-HEV antibodies based on gender, age, marriage and risk factors

	**Thalassemia patients (N=120/100%)**	**Anti HEV-Ab positive (N=2/1.67%)**
**Gender**		
Male	42 (35.0%)	2 (1.67%)
Female	78 (65.0%)	0 (0.00%)
**Age groups**		
0–20	9 (7.50%)	0.0%
21–30	34 (28.33%)	0.0%
31–40	66 (55%)	2 (1.67%)
41–50	11 (9.17%)	0 (0.00%)
**Marriage**		
Single	75 (62.50%)	2 (1.67%)
Married	45 (37.50%)	0 (0.00%)
**Risk Factors**		
History of surgery	60 (50.00%)	0 (0.00%)
History of hospital admission	55 (45.83%)	2 (1.67%)
History of blood transfusion	79 (65.83%)	0.0%

The results of ELISA showed that 2 out of 120 serum samples were positive for anti- HEV Ab and 118 (98.33%) samples were negative. The overall seroprevalence of HEV in our population was 1.67%. Based on analyzing results of ELISA IgG and IgM with regards to baseline data (sex, age, and marital status) and the risk factors such as blood transfusion, there is no statistically significant difference (P> 0.05). Also, the findings indicated that none out of 120 of samples were positive for HEV RNA by Nested RT- PCR.

## DISCUSSION

HEV infection has a widespread distribution in different parts of the world (6, 10). Iran is considered as an endemic area for hepatitis E infection with seroprevalence rate above 5% in general population (28–29). Due to regular blood transfusion, multi transfusion thalassemia patients are prone to infection with viral hepatitis agents such as HCV and HEV (30, 31). Seroprevalence of HEV in our study was 1.67% which is less than HEV seroprevalence rate in Iranian general population. Previous studies have reported different seroprevalence rates of HEV infection in thalassemia patients. In study on 125 thalassemia patients, anti-HEV IgG was confirmed in 2.4% of them and no significant association was found between anti-HEV and anti-HCV in thalassemia patients (32). Another study in Jahrom, southern Iran, indicated that the prevalence of HEV IgM and IgG antibodies in thalassemia patients were 1.8% and 10%, respectively (31). The seroprevalence of anti-HEV antibodies in thalassemia patients in Mashhad, Iran, was 2.6% (33). The seroprevalence of HEV in healthy Iranian population is lower than neighbor countries that due to the significant rise in the level of public awareness, hygiene, and sanitation. In a study in Saudi Arabia, the prevalence of hepatitis E virus antibody in patients with beta-thalassemia major was 10.7%. This result showed that there is possibility of blood-borne HEV in the Saudi population (34).

Chronic hepatitis C may found in the history of thalassemia patients (1, 30). Approximately 80% of the adult thalassemia patients in the world are infected with HCV (30). It has been reported that the seroprevalence of HCV infection among thalassemia patients in different regions of Iran ranged from 2% to 32% (25). Accordingly, Fazel H et al. reported that the prevalence of HEV IgG antibodies in HCV infected patients was high with the rate of 9.4% in Jahrom, Southern Iran (11). Also, Keyvani et al. revealed that the seroprevalence of anti-HEV antibody in HCV infected patients was 7% that in comparison to Iranian blood donors was high (15). On the other hand, other studies in Iran reported the low seroprevalence of HEV among hemophilia and thalassemia patients compared to the general population (30). Elizee et al. reported that the seroprevalence of HEV among Iranian thalassemia patients with chronic hepatitis C was 1.6% (30) that is similar to our data in this study (1.67%).

In this study, none of samples were positive for HEV RNA by Nested RT- PCR. There is a limited study regarding HEV-RNA prevalence among blood donors in Iran. During the asymptomatic viremic phase of infection; only the existence of HEV- RNA in blood of the healthy blood donors with a normal range of liver enzymes and negative serological markers are prone to transmission of HEV infection through blood and blood products (7, 9). So, in addition to the fecal-oral route, transmissions of HEV via blood transfusion is recently considered as a potential threat to blood safety (6, 7, 9) and should be considered especially for pregnant women, transplant recipients, and immune-compromised patients (10, 25).

Regarding the effect of age on seroprevalence of HEV infection, the majority of studies have indicated that the prevalence of anti-HEV antibodies increased with age and there were significant differences between the age groups. In a study by Farshadpour et al. on the prevalence of HEV among adults in the south-west of Iran, HEV seroprevalence reached the highest rate of 90.9% in 61 to 70-year-old age group (9). Mellgren et al. have reported that the prevalence of anti-HEV IgG was associated with higher age in patients with chronic hepatitis C virus infection (35). Also, in the study Alizee et al. has been published that the seroprevalence of anti-HEV IgG rising from 0% in subjects aged below 20 years to 12% in subjects over 40 years (30). Overall in Iran, the seroprevalence rate of HEV infection increases with age because of higher exposure of middle-aged and elderly subjects to HEV (29). In terms of gender, several studies reported that the HEV-antibodies seropositivity was not significant between males and females in Iran. However, Arbiza et al. mentioned that among subjects in non-endemic areas, middle and elderly males have the highest incident rate (6). In the current study, there were no significant differences between sex, age, and risk factors such as blood transfusion with HEV infection. However, the seroprevalence rates of HEV infection in males was (1.67%) higher than females (0.00%).

In a study conducted in Ahvaz city, the highly endemic area for HEV, Karun river which is contaminated with the city sewage considered as the water source of HEV infection (9). The other routes of transmission of viruses (HEV, TTV, SENV) like blood transfusion in Iran is unknown and the importance of it undetermined (7). In a study on SEN virus infection with high prevalence in three groups (thalassemic patients infected with HCV, thalassemic patients and HCV patients without a history of transfusion) showed that routes other than blood transfusion must be involved in transmitting viruses (36). Also, in another study on pediatric thalassemia patients and healthy volunteer children with high prevalence (64.4% and 24.4% respectively) of Torque teno virus (TTV), indicated that controls group, which did not have a history of blood transfusion, hepatitis, parenteral treatment, or any known diseases, were TTV positive and the possibility of infection of control group via non-parenteral routes was considered (37).

## CONCLUSION

Seroprevalence of HEV in our study group was 1.67% which is less than HEV seroprevalence rate in Iranian general population. Therefore, transmission of HEV infection via blood transfusion seems to be uncommon in Iran and the fecal-oral route can be the predominant mode of transmission in Iran; however, more studies are required to confirm this issue.

## References

[1] Hoofnagle JH Nelson KE Purcell RH. Hepatitis E. N Engl J Med 2012;367:1237–1244. 23013075 10.1056/NEJMra1204512

[2] Thomas HC Lok ASF Locarnini SA Zuckerman AJ. Viral Hepatitis, 4th Edition John Wiley and sons, Ltd; 2014 p. 431.

[3] Van der Poel WH Verschoor F Van der Heide R Herrera MI Vivo A Kooreman M Hepatitis E Virus sequences in swine related to sequences in humans, the Netherlands. Emerg Infect Dis 2001; 7:970–976. 11747723 10.3201/eid0706.010608 PMC2631914

[4] Vasickova P Psikal I Kralik P Widen F Hubalek Z Pavlik I. Hepatitis E virus: a review. Vet Med 2007;52:365–384.

[5] Johargy AK Mahomed MF Khan MM Kabrah S. Anti hepatitis E virus seropositivity in a group of male blood donors in Makkah, Saudi Arabia. J Pak Med Assoc 2013; 63:185–189. 23894892

[6] Mirazo S Ramos N Mainardi V Gerona S Arbiza J. Transmission, diagnosis, and management of hepatitis E: an update. Hepat Med 2014;6: 45–59. 24966702 10.2147/HMER.S63417 PMC4051621

[7] Taherkhani R Farshadpour F. Epidemiology of hepatitis E virus in Iran. World J Gastroenterol 2016; 22:5143–5153. 27298557 10.3748/wjg.v22.i22.5143 PMC4893461

[8] Ahmed A Ali AI Ghazal H Fazili J Nusrat S. Mystery of hepatitis E virus: recent advances in its diagnosis and management. Int J Hepatol 2015; 2015:872431. 25692043 10.1155/2015/872431 PMC4322671

[9] Farshadpour F Makvandi M. Prevalence of hepatitis E virus among adults in South-West of Iran. Hepat Res Treat 2015; 2015:759589. 26199756 10.1155/2015/759589 PMC4493289

[10] Lee GY Poovorawan K Intharasongkroh D Sa-nguanmoo P Vongpunsawad S Chirathaworn C Hepatitis E virus infection: Epidemiology and treatment implications. World J Virol 2015;4: 343–355. 26568916 10.5501/wjv.v4.i4.343 PMC4641226

[11] Fazel H Shadmand E Baharlou R Shokouh MR Hashemi SMA Farahmand M Sero-epidemiology of hepatitis E infections in patients with chronic hepatitis C virus infection in Jahrom, Southern Iran. Iran J Virol 2016; 10: 7–12.

[12] Ahmadi Ghezeldasht S Miri R Hedayatimoghadam M Shamsian A Bidkhori H Fathimoghadam F Population movement and virus spreading: HEV spreading in a pilgrimage city, Mashhad in Northeast Iran; an Example. Hepat Mon 2013; 13(8):e10255. 24171006 10.5812/hepatmon.10255 PMC3810681

[13] Saffar MJ Farhadi R Ajami A Khalilian AR Babamahmodi F Saffar H. Seroepidemiology of hepatitis E virus infection in 2–25-year-olds in Sari district, Islamic Republic of Iran. East Mediterr Health J 2009; 15:136–142. 19469436

[14] Joulaei H Rudgari O Motazedian N Gorji-Makhsous S. Hepatitis E virus seroprevalence in HIV positive individuals in Shiraz, Southern Iran. Iran J Microbiol 2015; 7:103–108. 26622971 PMC4662777

[15] Keyvani H Shamsi Shahrabadi M Najafifard S Hajibeigi B Fallahian F Alavian S. Seroprevalence of anti-HEV and HEV RNA among volunteer blood donors and patients with Hepatitis B and C in Iran. Bangladesh Liver J 2009; 1: 34–37.

[16] Karimi Elizee P Alavian SM Miri SM Behnava B Alavian SH Keshvari M The seroprevalence of entrically transmitted viral hepatitis in HCV infected thalassemia and hemophilia patients in Iran. Jundishapur J Microbiol 2013; 6(7): e9091.

[17] Lavanchy D. Evolving epidemiology of hepatitis C virus. Clin Microbiol Infect 2011; 17:107–115. 21091831 10.1111/j.1469-0691.2010.03432.x

[18] Sarkari B Eilami O Khosravani A Sharifi A Tabatabaee M Fararouei M. High prevalence of hepatitis C Infection among high risk groups in Kohgiloyeh and Boyerahmad Province, Southwest Iran. Arch Iran Med 2012;15:271–274. 22519374

[19] Zare F Fattahi MR Sepehrimanesh M Safarpour AR. Economic burden of Hepatitis C virus infection in different stages of disease: A report from southern Iran. Hepat Mon 2016; 16(4):e32654. 27257424 10.5812/hepatmon.32654 PMC4887962

[20] Mahmud S Akbarzadeh V Abu-Raddad LJ. The epidemiology of hepatitis C virus in Iran: Systematic review and meta-analyses. Sci Rep 2018;8:150. 29317673 10.1038/s41598-017-18296-9 PMC5760657

[21] Ataei B Hashemipour M Kassaian N Hassannejad R Nokhodian Z Adibi P. Prevalence of anti HCV infection in patients with beta-thalassemia in Isfahan-Iran. Int J Prev Med 2012; 3(Suppl 1):S118–123.. 22826753 PMC3399295

[22] Wonke B Hoffbrand AV Brown D Dusheiko G. Antibody to Hepatitis C virus in multiple transfused patients with thalassemia major. J Clin Pathol 1990; 43:638–640. 2119395 10.1136/jcp.43.8.638 PMC502643

[23] Jafroodi M Davoudi-Kiakalayeh A Mohtasham-Amiri Z Pourfathollah AA Haghbin A. Trend in prevalence of Hepatitis C virus infection among β-thalassemia major patients: 10 years of experience in Iran. Int J Prev Med 2015; 6: 89. 26445636 10.4103/2008-7802.164832 PMC4587076

[24] Marengo-Rowe AJ. The thalassemias and related disorders. Proc (Bayl Univ Med Cent) 2007;20:27–31. 17256039 10.1080/08998280.2007.11928230 PMC1769530

[25] Mirmomen SH Alvian SM. Treatment of HCV infection in multitransfused thalassemic patients: Does liver iron status affect the outcome of response? Hepat Mon 2005;5:11–13.

[26] Alavian SM Adibi P Zali MR. Hepatitis C virus in Iran: Epidemiology of an emerging infection. Arch Iranian Med 2005; 8: 84–90.

[27] Meng J Dubreuil P Pillot J. A new PCR-based seroneutralization assay in cell culture for diagnosis of hepatitis E. J Clin Microbiol 1997; 35:1373–1377. 9163446 10.1128/jcm.35.6.1373-1377.1997 PMC229751

[28] Mohebbi SR Rostami Nejad M Tahaei SM Pourhoseingholi MA Habibi M Azimzadeh P Seroepidemiology of hepatitis A and E virus infections in Tehran, Iran: a population based study. Trans R Soc Trop Med Hyg 2012;106:528–531. 22835757 10.1016/j.trstmh.2012.05.013

[29] Behzadifar M Bagheri-Lankarani K Abdi S Taheri Mirghaed M Beyranvand G Keshavarzi A Seroprevalence of hepatitis E virus in Iran: A systematic review and meta-analysis. Middle East J Dig Dis 2016; 8: 189–200. 27698968 10.15171/mejdd.2016.31 PMC5045671

[30] Alavian SM Miri SM Keshvari M Elizee PK Behnava B Tabatabaei SV Distribution of hepatitis C virus genotype in Iranian multiply transfused patients with thalassemia. Transfusion 2009; 49: 2195–2199. 19538541 10.1111/j.1537-2995.2009.02252.x

[31] Psichogiou M Tzala E Boletis J Zakopoulou N Loutradi A Maliori M Hepatitis E virus infection in individuals at high risk of transmission of non-A, non-B hepatitis and sexually transmitted diseases. Scand J Infect Dis 1996;28:443–445. 8953670 10.3109/00365549609037936

[32] Sotoodeh Jahromi A Ahmadi-Vasmehjani A Zabetian H Hakimelahi H Yusefi A Sanie MS Sero-epidemiological study of hepatitis E virus among thalassemia as high risk patients: A cross-sectional survey in Jahrom, Southern, Iran. Glob J Health Sci 2016; 8:53885. 27157168 10.5539/gjhs.v8n9p245 PMC5064080

[33] al-Fawaz I al-Rasheed S al-Mugeiren M al-Salloum A al-Sohaibani M Ramia S. Hepatitis E virus infection in patients from Saudi Arabia with sickle cell anaemia and beta-thalassemia major: possible transmission by blood transfusion. J Viral Hepat 1996;3:203–205. 8871882 10.1111/j.1365-2893.1996.tb00096.x

[34] Golshan A Abrishami F. Comparison between the frequencyof hepatitis E virus among major thalassemia patients with control group in Mashhad, North Eastof Iran. Int J Infect 2017; 4(4):e14180.

[35] Mellgren Å Karlsson M Karlsson M Lagging M Wejstål R Norder H. High seroprevalence against hepatitis E virus in patients with chronic hepatitis C virus infection. J Clin Virol 2017;88:39–45. 28160727 10.1016/j.jcv.2017.01.005

[36] Sani HO Sharifi Z Hosseini SM Shooshtari MM. SEN virus detection in thalassemic patients infected with hepatitis C virus. Arch Virol 2012;157:2441–2445. 22907824 10.1007/s00705-012-1443-3

[37] Alavi S Valeshabad AK Sharifi Z Nourbakhsh K Arzanian MT Navidinia M Torque teno virus and hepatitis C virus co-infection in Iranian pediatric thalassemia patients. Turk J Haematol 2012; 29:156–161. 24744647 10.5505/tjh.2012.20280 PMC3986954

